# CXCL12 defines lung endothelial heterogeneity and promotes distal vascular growth

**DOI:** 10.1242/dev.200909

**Published:** 2022-10-31

**Authors:** Prashant Chandrasekaran, Nicholas M. Negretti, Aravind Sivakumar, Derek C. Liberti, Hongbo Wen, Maureen Peers de Nieuwburgh, Joanna Y. Wang, Nigel S. Michki, Fatima N. Chaudhry, Sukhmani Kaur, MinQi Lu, Annabelle Jin, Jarod A. Zepp, Lisa R. Young, Jennifer M. S. Sucre, David B. Frank

**Affiliations:** ^1^ Department of Pediatrics, Division of Cardiology, University of Pennsylvania, Children's Hospital of Philadelphia, Penn-CHOP Lung Biology Institute, Penn Cardiovascular Institute, Philadelphia, PA 19104, USA; ^2^ Department of Pediatrics, Department of Cell and Developmental Biology, Vanderbilt University Medical Center, Nashville, TN 37232, USA; ^3^ Department of Medicine, University of Pennsylvania, Penn-CHOP Lung Biology Institute, Philadelphia, PA 19104, USA; ^4^ Department of Pediatrics, Division of Pulmonary and Sleep Medicine, University of Pennsylvania, Children's Hospital of Philadelphia, Penn-CHOP Lung Biology Institute, Philadelphia, PA 19104, USA

**Keywords:** Lung, Endothelium, Single-cell RNA sequencing, Mouse

## Abstract

There is a growing amount of data uncovering the cellular diversity of the pulmonary circulation and mechanisms governing vascular repair after injury. However, the molecular and cellular mechanisms contributing to the morphogenesis and growth of the pulmonary vasculature during embryonic development are less clear. Importantly, deficits in vascular development lead to significant pediatric lung diseases, indicating a need to uncover fetal programs promoting vascular growth. To address this, we used a transgenic mouse reporter for expression of *Cxcl12*, an arterial endothelial hallmark gene, and performed single-cell RNA sequencing on isolated *Cxcl12*-DsRed^+^ endothelium to assess cellular heterogeneity within pulmonary endothelium. Combining cell annotation with gene ontology and histological analysis allowed us to segregate the developing artery endothelium into functionally and spatially distinct subpopulations. Expression of *Cxcl12* is highest in the distal arterial endothelial subpopulation, a compartment enriched in genes for vascular development. Accordingly, disruption of CXCL12 signaling led to, not only abnormal branching, but also distal vascular hypoplasia. These data provide evidence for arterial endothelial functional heterogeneity and reveal conserved signaling mechanisms essential for pulmonary vascular development.

## Teaser

**Summary:** Analysis of a *Cxcl12* transgenic reporter mouse reveals distinct gene enrichment that defines functional and spatially unique endothelial subtypes in the developing pulmonary arterial tree.

## INTRODUCTION

Transition of blood through the lungs for respiration is dependent on an intact and adequate pulmonary vascular tree. Disruption due to prematurity, congenital malformations, and injury results in a deficient vascular bed leading to sequelae such as edema, hypoxemia and pulmonary hypertension. Prevention of the long-term effects of these sequelae depends on efficient regeneration and re-initiation of vascular fetal programs to restore circulation and preserve gas exchange (Cao et al., 2016; Ding et al., 2011; Mammoto et al., 2019; McCulley et al., 2018; Rafii et al., 2015; Vila Ellis et al., 2020; Zhao et al., 2020). This coordinated repair effort is crucial for preventing significant morbidity and mortality. As such, studies identifying the cellular and molecular mechanisms of pulmonary vascular development may inform vascular regeneration following injury.

Growing evidence indicates that the endothelium of the pulmonary circulation is heterogeneous. Studies using single-cell RNA-sequencing analysis on the developing and adult lung have uncovered distinct endothelial subpopulations comprising arteries, veins, capillaries and lymphatics (Asosingh et al., 2021; Gillich et al., 2020; Guo et al., 2019; He et al., 2018; Negretti et al., 2021; Niethamer et al., 2020; Rodor et al., 2021; Saygin et al., 2020; Schupp et al., 2021; Travaglini et al., 2020; Vila Ellis et al., 2020; Zepp et al., 2021). In addition, insights from these studies suggest functional specialization of endothelium. In particular, alveolar capillaries are composed of two populations predicted to participate in respiration or regeneration after injury based on unique transcriptional profiles (Gillich et al., 2020; Niethamer et al., 2020; Vila Ellis et al., 2020). Unlike capillary endothelium, data are limited in the macrovasculature during development and in adult disease and homeostasis (Asosingh et al., 2021). Moreover, there are no data profiling subpopulations of arterial endothelium in the developing lung. Identification and characterization of distinct, functional arterial endothelial cell subtypes and their signaling mechanisms during development are necessary for understanding the processes of growth, branching, angiogenesis and vessel assembly for tissue regeneration and replacement.

To assess arterial heterogeneity in the lung, we isolated arterial endothelium based on a previously identified arterial marker, C-X-C motif chemokine 12 (CXCL12). CXCL12 promotes growth and chemoattraction of cells expressing one of two receptors, C-X-C chemokine receptor 4 (CXCR4), and an alternative receptor, atypical chemokine receptor 3 (ACKR3) (Kiefer and Siekmann, 2011). CXCL12 plays a crucial role in tissue vascularization (Das et al., 2019; Ivins et al., 2015; Tachibana et al., 1998; Takabatake et al., 2009; Xu et al., 2014), and deletion of CXCL12 or its receptors leads to abnormal pulmonary artery branching in development and impaired capillary regeneration in a pneumonectomy model in adult mice (Kim et al., 2017; Rafii et al., 2015). Using fluorescence-activated cell sorting (FACS) of endothelial cells (ECs) from lungs of *Cxcl12^DsRed^* reporter mice (Ding and Morrison, 2013), we performed single-cell RNA sequencing on 26,652 cells across development. Surprisingly, we found *Cxcl12* expression at low levels in the subsets of previously identified capillary ECs concomitant with high levels of expression in the macrovascular arterial ECs (maECs). Using unbiased clustering and cell annotation, we observed proximal and distal patterning of the arterial endothelium across development with unique gene enrichment consisting of genes associated with vascular wall assembly and angiogenesis, respectively. Finally, deletion of CXCL12 during development resulted in not only the previously identified aberrant arterial branching but also pulmonary vascular hypoplasia, indicating a crucial role for the CXCL12 signaling axis in pulmonary vascular assembly and development.

## RESULTS

### Morphometric analysis of the developing pulmonary arterial system

**Fig. 1.**
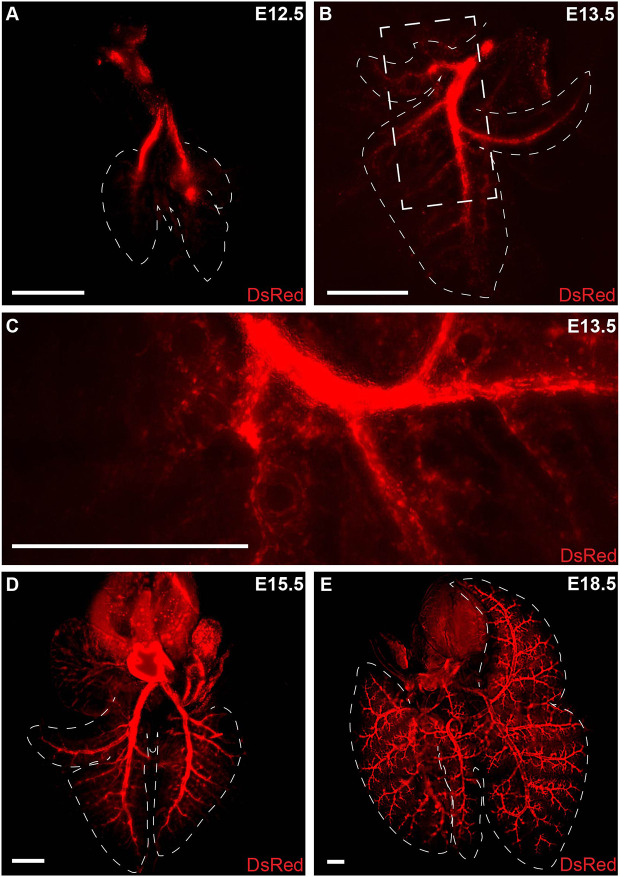
**Morphogenesis of the developing pulmonary arterial tree in *Cxcl12^DsRed/+^* reporter embryos.** (A) At E12.5, two intrapulmonary arteries are visible. (B) At E13.5, secondary branches extend out from the primary branches. (C) Higher magnification of secondary branching (boxed area in B). (D) At E15.5, increased secondary branching can be observed along with tertiary and quaternary branches. (E) At E18.5, robust and complex branching is apparent. Dashed lines delineate outlining of the lung lobes. Scale bars: 500 µm.

We performed an imaging analysis of the growing arterial tree throughout development to identify periods of rapid vascular growth and significant morphometric changes. Using the *Cxcl12^DsRed^* reporter mouse with *DsRed* knocked into exon 1 of the *Cxcl12* locus, we imaged whole lungs or single lobes of the developing arteries at embryonic day (E) 12.5, E13.5, E15.5 and E18.5 using confocal and Leica Thunder microscopy systems (Fig. 1). At several stages, we confirmed specificity of the reporter using immunohistochemistry (IHC) for DsRed reporter expression and markers for endothelium, including ETS-related gene (ERG) and endomucin (EMCN) (Fig. S1A-L). As in other organ systems, *Cxcl12*-DsRed is expressed highly throughout the arterial endothelium with additional expression observed in the mesenchyme, including arterial vascular smooth muscle cells (VSMCs) (Chang et al., 2017; Ghadge et al., 2021; Ivins et al., 2015; Takabatake et al., 2009). Of note, we observed no DsRed expression in venous endothelium during these developmental time periods (Fig. S1G,H,J). As early as E12.5, DsRed was expressed at high levels in the pulmonary arteries marking two primary intrapulmonary branches (Fig. 1A). One day later, we observed the initiation of secondary branching from the primary intrapulmonary arteries with small, thin, tubular structures appearing to sprout from the intrapulmonary artery and grow outward to the lung periphery (Fig. 1B,C). By E15.5, the intrapulmonary arteries evolved with the formation of additional secondary branches along with tertiary and quaternary arterial branches (Fig. 1D). Subsequently, the arterial tree underwent significant growth and further branching complexity until the end of prenatal vascular development (Fig. 1E).

### Single-cell transcriptomic analysis uncovers heterogeneity of the *Cxcl12*^+^ endothelium

**Fig. 2.**
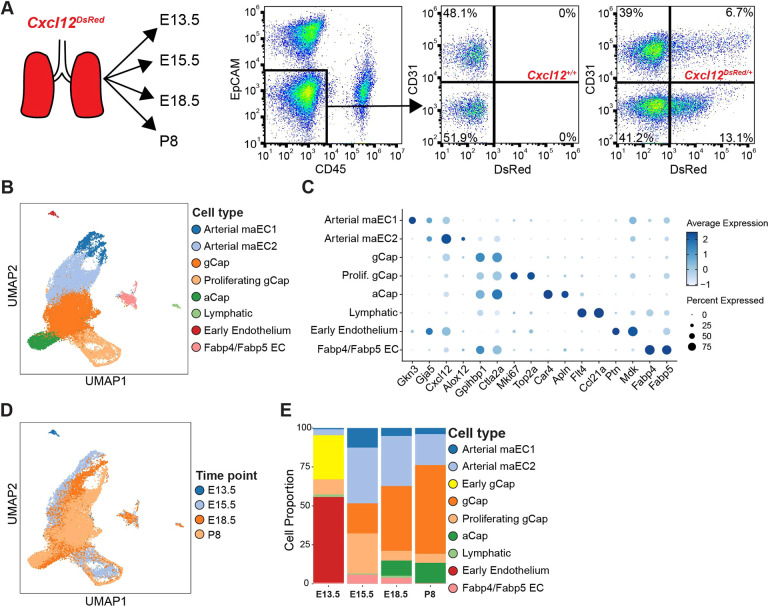
**Sequencing of *Cxcl12*^+^ cells uncovers heterogeneity and previously undefined arterial EC subpopulations.** (A) FACS strategy and plots for *Cxcl12*-DsRed^+^ cells. (B) UMAP embedding of *Cxcl12*-DsRed^+^ cells (*n*=26,652) colored by cell type. (C) Dot plot of marker genes for each cell type where dot size indicates the proportion of cells within a cluster expressing a gene, and color intensity indicates the relative expression level. (D) UMAP embedding colored by time point. (E) Cellular composition changes in *Cxcl12*^+^ ECs over time.

Using the data from our morphometric analyses, we selected specific developmental periods with significant changes in structure and growth to assess cellular and transcriptomic composition of the growing arterial tree. Single-cell suspensions were prepared from a mixture of two to four lungs acquired at E13.5, E15.5, E18.5 and postnatal day (P) 8. We obtained *Cxcl12*-DsRed+ ECs using a FACS-based strategy [DsRed^+^/CD31 (PECAM1)^+^/EpCAM^−^/CD45^−^] (Fig. 2A). Single-cell RNA sequencing (scRNA-seq) was performed using a droplet-based platform (10x Genomics Chromium). Across the four time points, a total of 26,652 endothelial single-cell transcriptomes were analyzed, with 391 cells at E13.5, 9082 cells at E15.5, 9016 cells at E18.5 and 8163 cells at P8 (Fig. 2B). The median unique molecular identifiers (UMIs) per cell was 6666 (interquartile range 6253-7545), and a median of 2483 genes (interquartile range 2059-3003) was detected per cell.

Louvain clustering was applied to the single-cell transcriptomes to identify transcriptionally related clusters, identifying eight cellular populations, including two previously unidentified arterial EC populations: arterial maEC1, marked by *Gkn3* and *Gja5* expression; arterial maEC2, marked by elevated *Cxcl12* and *Alox12* expression; general capillaries (gCaps), marked by *Gpihbp1* and *Ctla2a* expression; proliferating gCaps, marked by *Mki67* and *Top2a* expression; alveolar capillaries (aCaps), marked by *Car4* and *Apln* expression; lymphatic endothelium, marked by *Flt4* and *Ccl21a* expression; early endothelium, marked by *Ptn* and *Mdk*; and a cluster of endothelial cells marked by *Fabp4* and *Fabp5* expression (Fig. 2C, Table S1). Examining the uniform manifold approximation and projection (UMAP) by time indicated that E13.5 is the most distinct from the other time points (Fig. 2D,E). The other three time points (E15.5, E18.5 and P8) had relatively similar cellular composition except for the proliferating gCap cells, which decreased in abundance from E15.5 to E18.5 (Fig. 2D,E). Further analysis of RNA-velocity-based cellular transitions suggested potential transitions between arterial maEC1, arterial maEC2 and gCap populations (Fig. S2A). Although velocity analysis can be influenced by the underlying UMAP structure and should be interpreted with caution, this is consistent with other findings (Zheng et al., 2022 preprint). To assess the genes that share expression patterns with *Cxcl12*, a Spearman's rank correlation coefficient was calculated between every gene and *Cxcl12*. There were 23 genes with a correlation coefficient greater than 0.3, including *Gja4* and *Slc6a6* (Fig. S2B). In addition, we confirmed that DsRed transcript expression overlapped with *Cxcl12* across all time points (Fig. S2C).

### Temporal allocation of CXCL12 endothelium reveals distinct spatial and functional populations

**Fig. 3.**
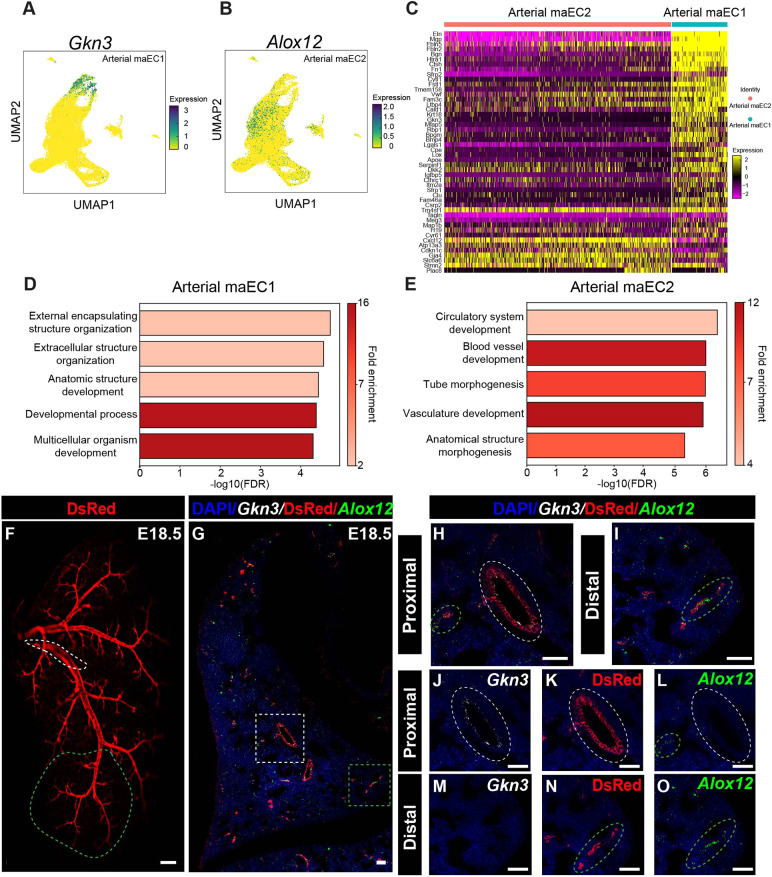
**Identification of proximal and distal arterial ECs by RNA FISH and predicted function analysis.** (A) UMAP embedding of cells colored by *Gkn3* expression. (B) UMAP embedding of cells colored by Alox12 expression. (C) Heat map representing expression of hallmark genes in arterial macro endothelial cell types 1 and 2 (arterial maEC1 and maEC2). (D) GO analysis of the maEC1 cluster. (E) GO analysis of the maEC2 cluster. (F) Whole-mount image showing Cxcl12-DsRed arterial endothelium. Dashed white line marks the proximal artery branch and the dashed green line marks the arterial tree from the distal tip of the secondary branch to the quinary branches. (G) RNA FISH for *Gkn3* and *Alox12* and IHC for DsRed protein at E18.5 proximal (white box) and distal (red box) regions. (H) Higher magnification of RNA FISH for *Gkn3* and *Alox12* and IHC for DsRed protein in the proximal region. (I) Higher magnification of RNA FISH for *Gkn3* and *Alox12* and IHC for DsRed protein in the distal region. (J-L) Separate channels for proximal region RNA FISH for *Gkn3* (J), IHC staining for DsRed (K) and *Alox12* (L). (M-O) Separate channels for proximal region RNA FISH for *Gkn3* (M), IHC staining for DsRed (N) and *Alox12* (O). Scale bars: 50 mm (F); 50 μm (G-O).

Analysis of transcriptomic data identified two subpopulations of maECs that persist throughout development (Fig. 2B), and we have identified *Gkn3* and *Alox12* specifically defining these clusters in arterial maEC1 and maEC2 subpopulations, respectively (Fig. 3A,B). GKN3 has no previously described role in lung development, but it has been identified as a possible receptor for the Japanese Encephalitis Virus (Mukherjee et al., 2018) and as an arterial endothelial marker in the brain (Vanlandewijck et al., 2018). ALOX12 is an enzyme involved in arachidonic acid metabolism that promotes tumor progression and angiogenesis (Zheng et al., 2020). Importantly, ALOX12 has also been implicated in hypoxia-induced endothelial angiogenesis and smooth muscle cell proliferation in the lung (Preston et al., 2006; Zhang et al., 2018). To characterize functional differences between these two populations, we generated heatmaps to interrogate differential gene expression of the top 40 positively associated marker genes for each cluster and used Panther Gene Ontology (GO) enrichment analysis to predict potential biological functional differences based on differential gene expression for each population (Fig. 3C-E, Table S2). Although there was some overlap in the functions identified, we noted several distinct predicted roles for each group. For maEC1, the top representative genes predicted roles in extracellular matrix and structure organization, external encapsulating structure organization, and multicellular organism development (Fig. 3D). These genes included *Eln*, *Mgp*, *Fbln5*, *Fbln2*, *Fn1* and *Lox*, suggesting a function in vessel wall assembly. The arterial maEC2 subpopulation expressed many genes associated with vascular development as noted by GO analysis (Fig. 3E). Genes included *Jag1*, *Fbn1*, *Sox17*, *Gata6*, *Cxcl12*, *Hey1*, *Tgfb2* and *Vegfc*.

The distinct functional attributes suggested by gene expression and ontology analysis point to spatially defined arterial endothelial compartmentalization. Given that arterial maEC2s were enriched in genes involved in vascular development and in a spatial continuum with capillary ECs on UMAP-based clustering, we hypothesized that they represent a distal arterial maEC (distal maEC2) population. In addition, arterial maEC1 had gene enrichment in extracellular structure, suggesting a functional role in vessel wall assembly. Combined with their spatial location further away from capillary ECs on UMAP-based clustering, we hypothesized that they represent a proximal arterial maEC (proximal maEC1) population. To assess the spatial localization of these subpopulations, we performed RNA *in situ* hybridization (RNA FISH) for *Alox12* and *Gkn3* combined with IHC for DsRed in *Cxcl12^DsRed^* reporter embryonic lung tissue at E15.5 (Fig. S3A-M) and E18.5 (Fig. 3F-O). At both time points, *Alox12* localized to the distal arterial endothelium with no expression in the more proximal arteries (Fig. 3G,I,L,O, Fig. S3C,D,G,J,M). By contrast, *Gkn3* was expressed proximally with extension limited peripherally (Fig. 3G,H,J,M, Fig. S3A,B,E,H,K). Both distal Alox12^+^ and proximal Gkn3^+^ arterial EC populations colocalized with *Gja5*, confirming specificity to the arterial ECs (Fig. S3N-Q). In addition, *Gkn3* endothelial expression accompanied expression of both actin alpha 2 (ACTA2) and tropoelastin (ELN), supporting a role in endothelial-mediated vessel wall assembly (Fig. S3R,S).

Interestingly, scRNA-seq of the *Cxcl12*-DsRed^+^ endothelium included not only arterial endothelium but also capillary endothelium (Fig. 2B). Although *Cxcl12* expression and DsRed expression were low in these populations, all previously described capillary EC populations were identified, including gCap, proliferating gCap, and aCap cells, marked by expression of *Gpihbp1*, *Mki67*, and *Apln* or *Car4*, respectively (Fig. S2C). The gCap and aCap subpopulations have been recently described as capillary 1 cells (CAP1) and capillary 2 cells (CAP2) (Sun et al., 2022). In concordance with previous data, in our study both differentiated gCap and aCap populations arose after E15.5 and were apparent by E18.5 (Gillich et al., 2020; Negretti et al., 2021; Vila Ellis et al., 2020; Zepp et al., 2021). We validated the transcriptional profile and localized these populations at the tissue level with RNA FISH for *Cxcl12*, *Apln* and *Gpihbp1* transcripts (Fig. S3T,U). Again, expression of *Cxcl12* transcript resided predominantly in the arterial endothelium. At higher magnification, we observed rare transcript expression in capillary endothelium.

### Characterization of CXCL12 expression and its signaling axis

**Fig. 4.**
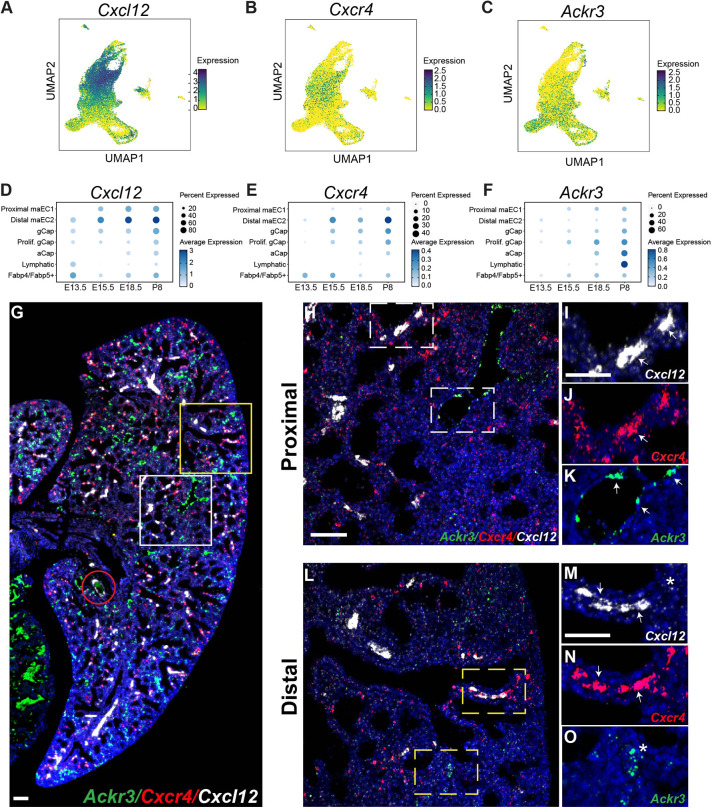
**Characterization of *Cxcl12* expression and its signaling axis.** (A-C) UMAP embedding of cells colored by *Cxcl12* (A), *Cxcr4* (B) and *Ackr3* (C) expression. (D-F) Dot plots of *Cxcl12* (D) *Cxcr4* (E) and *Ackr3* (F) expression in different EC compartments at E13.5, E15.5, E18.5 and P8. (G) RNA FISH for spatial expression of *Ackr3*, *Cxcr4* and *Cxcl12* in ECs and VSMCs at E18.5. The red circle denotes a proximal artery. (H) Higher magnification of RNA FISH for *Ackr3*, *Cxcr4* and *Cxcl12* in proximal lung at E18.5 (white boxed area in G). (I-K) Higher magnification of the boxed areas in H. (L) Higher magnification of RNA FISH for *Ackr3*, *Cxcr4* and *Cxcl12* in distal lung at E18.5 (yellow boxed area in G). (M-O) Higher magnification of boxed areas in L. Arrows indicate expression in macrovessels. Asterisks indicate expression in capillaries. Scale bars: 100 µm (G); 50 µm (H-O).

Low *Cxcl12* expression in the distal capillary plexus and high expression in the arterial tree indicated a gradient of *Cxcl12* expression. We performed UMAP embedding of *Cxcl12*-DsRed^+^ ECs confirming the presence of differential expression of *Cxcl12* across endothelial subpopulations (Fig. 4A,D). From this, we predicted a spatial CXCL12 gradient in the tissue, and we determined the EC subpopulations expressing the CXCL12 receptors CXCR4 and ACKR3. UMAP embedding of *Cxcl12*-DsRed^+^ endothelium interrogated for *Cxcr4* expression indicated a cellular distribution to the distal maEC2 cells and a small portion of the capillary ECs (Fig. 4B,E). Similar embedding by *Ackr3* confined expression to capillary ECs (Fig. 4C,F). Because developing *Cxcl12*-DsRed^+^ endothelium was void of venous identity in the lung, we reanalyzed previously published scRNA-seq data of developing mouse lung endothelium for the CXCL12 signaling axis distribution (Negretti et al., 2021). Although not as distinct, we observed segregation of the arterial ECs into proximal and distal subpopulations (Fig. S4A). Whereas *Cxcr4* remained localized to arterial and capillary endothelium, *Ackr3* was expressed not only in the capillary endothelium but also robustly in venous endothelium (Fig. S4A-C,E,F) throughout prenatal and postnatal lung development. In addition, C*xcl12* became more broadly expressed in the developing postnatal lung and could be observed in a small number of venous ECs (Fig. S4D,G).

We performed spatial and temporal validation of expression of the CXCL12 signaling axis using RNA FISH on E15.5 and E18.5 C*xcl12^DsRed/+^* embryonic tissue. *Cxcr4* and *Ackr3* expression was concomitantly determined with *Cxcl12* expression. *Cxcr4* is expressed in the distal vasculature overlapping with robust *Cxcl12* expression (Fig. 4G-O). In addition, a scattering of capillary ECs contained *Cxcr4* (Fig. 4J,N). Similarly, *Ackr3* localized to capillary ECs, but it was more prominent in the venous endothelium (Fig. 4K,O). Ackr3 and Cxcr4 were not colocalized in the distal vasculature. Although there was significant overlap of *Cxcr4* and *Cxcl12* in the distal maEC2s, *Cxcl12* and *Ackr3* were colocalized to a lesser degree, with *Ackr3* detected almost exclusively in the capillary and venous endothelium (Fig. S4H-K).

### Global loss of CXCL12 results in distal branching defects and pulmonary vascular hypoplasia

**Fig. 5.**
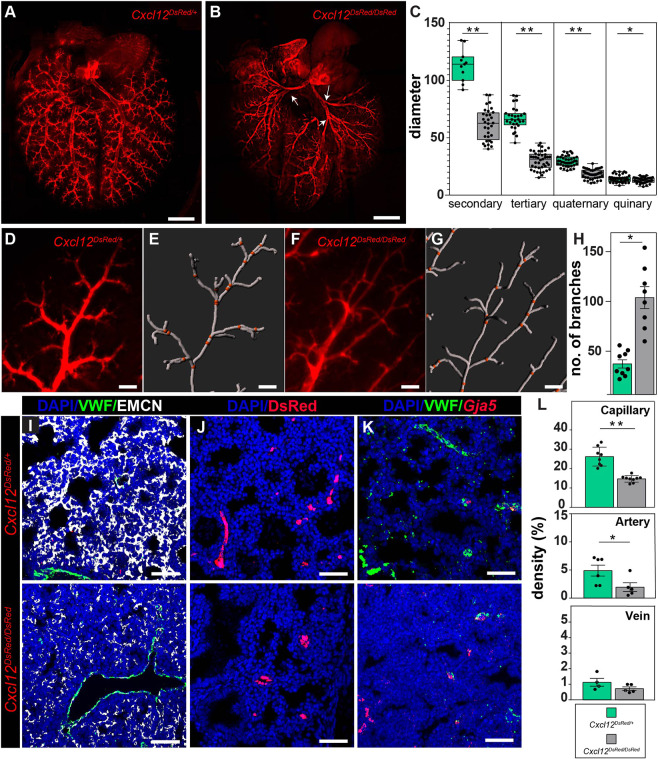
**Loss of *Cxcl12* leads to branching defects and pulmonary vascular hypoplasia.** (A,B) Whole-mount imaging of arterial endothelium in *Cxcl12^DsRed/+^* (A) and *Cxcl12^DsRed/DsRed^* (B) at E18.5. *Cxcl12^DsRed/DsRed^* lungs show defective proximal branching (white arrows). (C) Arterial diameter distribution of control *Cxcl12^DsRed^* versus null *Cxcl12^DsRed/DsRed^* arteries (*n*=4, filaments measured ≥10, **P<0.005, *P<0.05). (D-H) Branching analysis using Imaris Filament. (D,E) Representative image of *Cxcl12^DsRed/+^* artery (D) and Filament tracing using IMARIS 9.2 (E). (F,G) Representative image of *Cxcl12^DsRed/DsRed^* artery (F) and Filament tracing (G). (H) Number of branches from the quaternary branch in the periphery (*n*=4, images analyzed ≥8, *P<0.05). (I-L). Vascular density measurements in control *Cxcl12^DsRed^* (top) versus null *Cxcl12^DsRed/DsRed^* (bottom) lung tissue. (I) Capillary density IHC staining of tissue sections for VWF (macrovessel) and EMCN (capillary and vein). (J) Arterial density IHC staining of tissue sections for DsRed (artery). (K) Venous density IHC and RNA FISH staining for *Gja5* (artery) and VWF (macrovessel) expression. (L) Volume density calculations of capillaries, arteries and veins (*n*≥4, **P<0.005, *P<0.05). Scale bars: 1 mm (A,B); 100 µm (D-G); 50 µm (I-K).

Expression and localization of the CXCL12 signaling axis indicates a prominent role in the formation of the distal vasculature. To disrupt CXCL12 signaling, we generated *Cxcl12* heterozygous (*Cxcl12^DsRed/+^*) controls and *Cxcl12* homozygous null (*Cxcl12^DsRed/DsRed^*) embryos. As previously reported (Kim et al., 2017), no postnatal *Cxcl12^DsRed/DsRed^* were recovered. Whole-mount immunofluorescence imaging was performed first to assess morphological changes in the microvasculature at E18.5. Consistent with previously published data (Kim et al., 2017), we observed proximal and distal branching defects (Fig. 5A,B). In addition, we noted diminished mean vessel diameter for secondary to quinary branches in null embryos (Fig. 5C). Distally, the arteries lacked directionality toward the periphery, which was preserved in control embryos (Fig. S5A,B). Quantification of branching angles of the distal arteries using Filament Tracing in Imaris revealed discrepant angles in *Cxcl12^DsRed/DsRed^* embryos compared with controls (Fig. S5C). Additionally, Sholl analysis (Sholl, 1953), measuring branching complexity by quantification of vessel intersections of drawn concentric shells from the secondary branch, was abnormally high in *Cxcl12^DsRed/DsRed^* embryos compared with controls (Fig. S5D,E). Furthermore, *Cxcl12^DsRed/DsRed^* embryonic lungs appeared to have an increase in total branching number, indicating a compensatory mechanism to increase vessel density (Fig. 5D-H). Although null embryos contain two copies of the DsRed allele that could potentially increase the visualization of the reporter, flow cytometric analysis of DsRed^+^ endothelium revealed no differences in fluorescence brightness in the heterozygous versus homozygous null embryonic lungs (Fig. S6A).

CXCL12 and its receptors, CXCR4 and ACKR3, were expressed in a compartmental fashion. Thus, deficits in the vascular bed may expand beyond the arterial endothelium. To quantify arterial, venous and capillary endothelium, we performed IHC and RNA FISH for multiple combinations of markers of arterial, venous and capillary endothelium. We used von Willebrand factor (VWF) to identify macrovessels, *Gja5* and DsRed for arterial endothelium, neuregulin 1 (NRG1) for venous endothelium, and EMCN for capillary and venous endothelium in lung tissue sections at E18.5 (Fig. 5I-L, Fig. S5F-I). Using 3D surface rendering, we were able to quantify the vessel density in tissue sections by calculating the percentage volume of capillary, artery or vein in each section (Chen et al., 2021; Nizari et al., 2019). The majority of the vessel density consisted of capillaries, and capillary density was significantly reduced in the *Cxcl12^DsRed/DsRed^* embryos compared with controls (Fig. 5I,L, Fig. S5F). Similarly, arterial density was modestly reduced, but the density of veins was unchanged (Fig. 5J-L, Fig. S5G,H). We also calculated the total number of macrovessels in a tissue section as branching was increased in *Cxcl12^DsRed/DsRed^* embryos (Fig. S5I). Although there was a trend toward an increase in arterial number, it was not statistically significant (Fig. S5I). However, we did note a significant decrease in the number of veins.

**Fig. 6.**
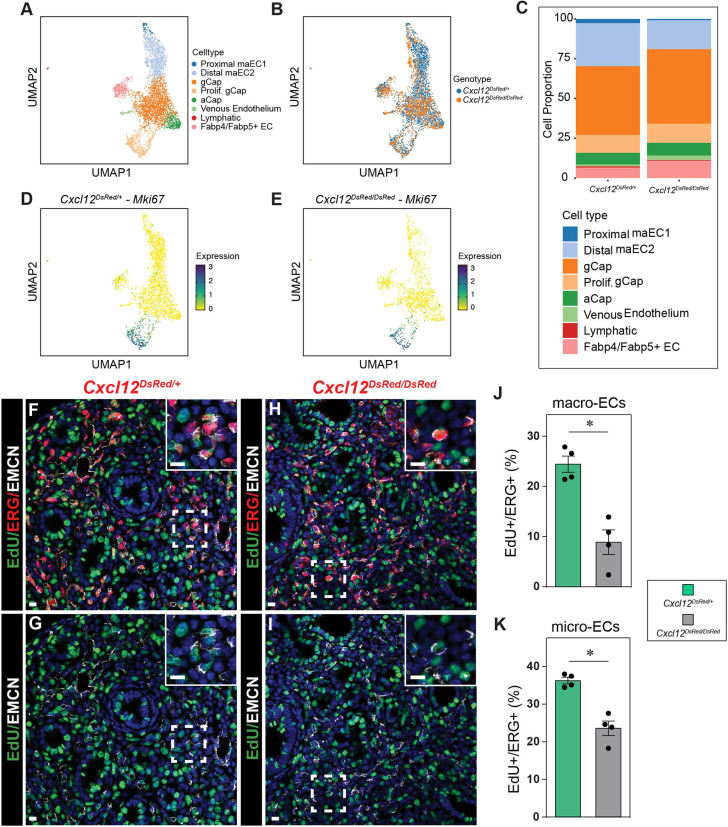
**Decreased proliferation in control *Cxcl12^DsRed^* versus null *Cxcl12^DsRed/DsRed^* ECs.** (A) UMAP embedding of cells colored by endothelial cell populations at E18.5. (B) UMAP embedding of cells colored by *Cxcl12^DsRed/+^* and *Cxcl12^DsRed/DsRed^* ECs at E18.5. (C) Proportion of different cell types in *Cxcl12^DsRed/+^* and *Cxcl12^DsRed/DsRed^* at E18.5. (D,E) UMAP embedding of cells colored by *Mki67* expression in *Cxcl12^DsRed/+^* (D) and *Cxcl12^DsRed/DsRed^* (E) ECs at E18.5. (F-K) Assessment of proliferation measured by EdU incorporation in *Cxcl12^DsRed/+^* and *Cxcl12^DsRed/DsRed^* macro- and micro-ECs. (F-I) IHC for EdU, ERG and EMCN to mark proliferating macro- and micro-ECs in in *Cxcl12^DsRed/+^* (F,G) and *Cxcl12^DsRed/DsRed^* (H,I) lung sections. Insets show higher magnification of the boxed areas. (J,K) Quantification of the percentage of EdU^+^/ERG^+^ macro- (J) and micro- (K) ECs in *Cxcl12^DsRed/+^* and *Cxcl12^DsRed/DsRed^* lungs (*n*=4). *P<0.005. Scale bar: 10 µm (F-I, main panels); 10 µm (F-I, insets).

To corroborate our quantitative findings and ascertain affected cell populations, we performed scRNA-seq on whole E18.5 lungs from control and *Cxcl12^DsRed/DsRed^* embryos (Fig. S6A-C). Overall, we saw that the percentage of ECs in the lung was diminished (Fig. S6D). We extracted the EC data and re-clustered to examine changes in the cellular representation of the endothelium (Fig. 6A,B). From these data, we were able to re-identify all previously referenced EC clusters.

To determine whether differences in proliferation are a mechanism responsible for the diminished number of ECs and pulmonary hypoplasia, we examined *Mki67* expression, noting no significant changes in the number of proliferating ECs at E18.5 by scRNA-seq (Fig. 6C-E). We further interrogated proliferation by IHC at E15.5, an earlier time point when the vasculature demonstrates more rapid growth. Using antibodies to EMCN to mark capillary and venous endothelium, ERG for pan-endothelium, and 5-ethynyl-2′-deoxyuridine (EdU) incorporation for proliferation, we quantified the number of proliferating endothelial cells (Fig. 6F-K). At E15.5, we observed a significant decrease in the proliferation of both proximal macrovessel and distal plexus ECs in *Cxcl12^DsRed/DsRed^* embryonic lungs, indicating an early proliferative defect.

### Endothelial loss of CXCL12 leads to primary branching defects in the pulmonary artery

**Fig. 7.**
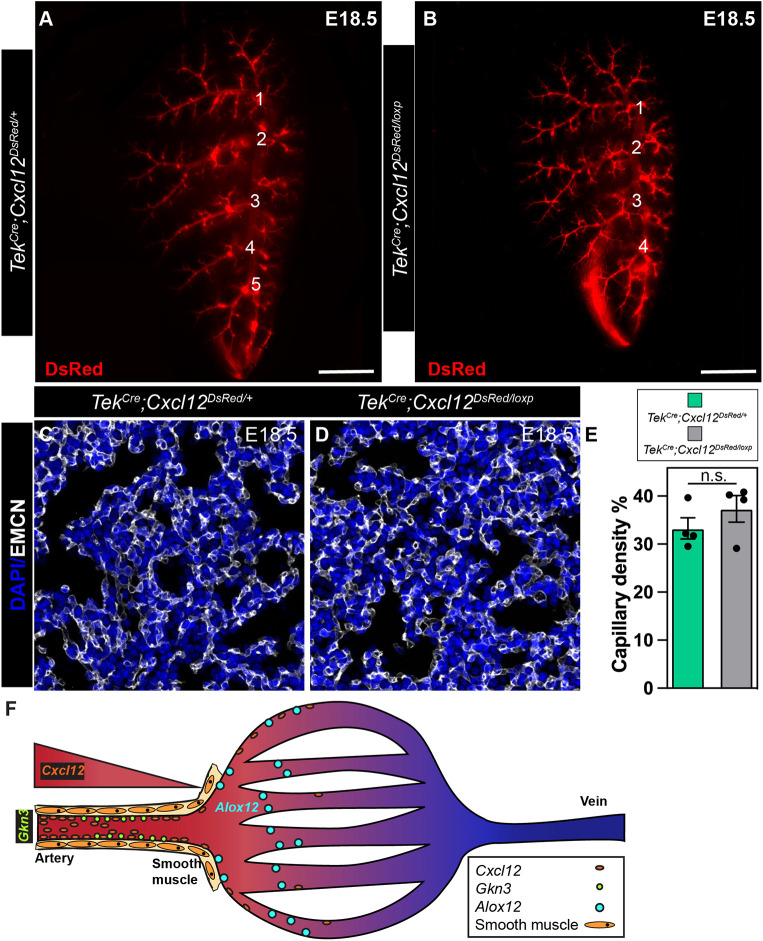
**EC-specific loss of CXCL12 leads to branching defects.** (A,B) Whole-mount imaging of the left lobe of *Tek^Cre^;Cxcl12^DsRed/+^* (A) and *Tek^Cre^;Cxcl12^DsRed/loxp/+^* (B) embryos (numbers signify branching points). (C,D) IHC staining for EMCN to delineate capillaries in *Tek^Cre^;Cxcl12^DsRed/+^* (C) and *Tek^Cre^;Cxcl12^DsRed/loxp/+^* (D) embryonic lungs. (E) Quantification of capillary density (*n*=4). n.s., not significant (*P*=0.485). (F) Model of CXCL12 and arterial heterogeneity. A gradient of *Cxcl12* expression exists in distal pulmonary vasculature that delineates the arterial endothelium into proximal *Gkn^+^* and distal *Alox12^+^* subpopulation with distinct functions. Scale bars: 1 mm (A,B); 50 µm (C,D).

CXCL12 is expressed highly not only in the distal arterial endothelium but also in the proximal vascular smooth muscle in the lung. To determine whether the phenotype exhibited by *Cxcl12^DsRed/DsRed^* is primarily endothelial driven, we deleted endothelial-derived CXCL12 using *Tek^Cre^* and assessed vascular development (Fig. 7A-E). Compared with control embryonic lungs, we observed primary branching defects in *Tek^Cre^;Cxcl12^DsRed/loxp^* embryonic lungs, wherein secondary branch number was decreased (Fig. 7A,B). Otherwise, the lung lobe looked relatively normal with subtle defects. To evaluate changes in capillary density, we performed IHC for EMCN and used 3D surface rendering to calculate the volume density (Fig. 7C,D). Our quantifications did not show any significant changes in capillary density (Fig. 7E). Although endothelial-derived CXCL12 contributes to some aspects of pulmonary vascular development, there likely exists synergistic or compensatory contribution(s) from additional CXCL12 signaling niches.

### Loss of CXCL12 leads to significant transcriptome changes in the endothelial niche

Lung development is a coordinated process involving communication between cellular and tissue types. Failure of this reciprocal behavior results in transcriptional changes and defects in development. This process can be elegantly annotated using scRNA-seq. Analysis of UMAP embedding of cells from two E18.5 control and two *Cxcl12^DsRed/DsRed^* embryonic lungs revealed transcriptional changes in multiple endothelial niche compartments (Fig. S6B,C, Table S3). Although overall numbers of cell populations were mostly unaffected, there were significant differences in cell type-specific transcriptomes in control versus null embryos. In particular, the immune cells, including the interstitial and alveolar macrophages, demonstrated significant changes in gene enrichment. In addition, the examination of the epithelium, including secretory, ciliated, alveolar type 1 (AT1) and alveolar type 2 (AT2) cells, revealed significant differences in gene enrichment (Fig. S6E-H). Evaluating broad changes using the top upregulated and downregulated genes in knockout versus control whole lungs was performed with GO term enrichment. We observed that there was an enrichment of genes associated with blood vessel morphogenesis and categories associated with mitochondrial bioenergetics in endothelial cells. The epithelium had enrichment of genes involved in the regulation of endothelin production. Immune cells expressed genes involved in microglial cell or macrophage activation, and mesenchymal cells were enriched in genes involved in membrane transport (Fig. S6E-H, Table S4).

Given that global deletion of CXCL12 led to multicompartmental transcriptomic changes in the lung, we assessed whether these changes were accompanied by defects in the non-endothelial CXCL12 signaling niche. Previous studies demonstrated CXCL12 signaling involvement in vascular smooth muscle (VSM) responses in development and disease (Dai et al., 2018; Stratman et al., 2020; Wei et al., 2015; Yuan et al., 2020). To assess CXCL12 effects on VSMCs during lung development, we performed a previously described method using IHC for ACTA2 and quantification of VSM wall thickness (Stratman et al., 2020). Quantification of VSM wall thickness surrounding proximal arteries revealed no differences between control and null embryonic lungs (Fig. S7A-C). Additionally, loss of CXCL12 led to significant transcriptome changes in the epithelium, indicating potential disruption of epithelial growth and development. We did not observe any obvious defects in epithelial branching morphogenesis and quantification of proliferating NKX2-1^+^ epithelial cells at E15.5, a time point showing significant epithelial and endothelial proliferation, revealed no significant difference (Fig. S7D-F) (Frank et al., 2016).

## DISCUSSION

We have developed a temporal and spatially integrated transcriptomic atlas of *Cxcl12*^+^ endothelium during lung development. With these data, we identified two arterial endothelial cells populations in the developing lung (Fig. 7F). This is one of the first studies uncovering functionally and spatially defined subpopulations in developing arterial endothelium. This distinction improves recognition of distinct roles for arterial ECs in patterning and assembly of the developing artery. In addition, we have delineated CXCL12 signaling components in the developing pulmonary vasculature and uncovered unique cell type-specific expression patterns. Finally, we have provided a framework for analysis of branching abnormalities in the pulmonary vasculature and uncovered several roles for CXCL12 in pulmonary vascular development, identifying a role in proliferation and vascular branching for the lung.

### Proximal-distal patterning of the pulmonary artery indicates distinct spatial functions

From E15.5 to P8, the intrapulmonary artery segregates into proximal and distal subpopulations. Gene set enrichment on GO terms in these populations suggests distinct functions. The proximal artery expresses genes associated with extracellular structure and matrix formation, suggesting a role for vessel wall construction. In contrast, the distal artery expresses genes important in vascular development and morphogenesis. Previous descriptions of distinct subpopulations of arterial endothelium in other developing organs, such as the coronary arterial system, have been limited to only subtypes of varying maturation stages (Su et al., 2018). Although compartmentalization of the capillary endothelium of the lung has been previously demonstrated, this is one of the first studies defining the developing arterial endothelium into spatial and functionally different subpopulations in any organ system (Gillich et al., 2020; Niethamer et al., 2020; Vila Ellis et al., 2020).

Functionally distinct arterial endothelial subpopulations exist in other adult organs as well. The kidney contains 24 subpopulations of endothelium, and arterial endothelium can be divided into efferent, afferent and juxtaglomerular apparatus-associated afferent arteriole populations by gene expression and function (Dumas et al., 2020). These three populations have previously established physiological functions within the kidney. Similarly, the aorta is segregated into subpopulations that regulate different arterial responses at homeostasis and in hypertensive states in humans and mice (Kalluri et al., 2019; Zhang et al., 2021). Similar findings exist in a study on the adult human pulmonary artery endothelium in health and disease. The pulmonary artery endothelium has been profiled from adult control and patients with pulmonary arterial hypertension (PAH) (Asosingh et al., 2021). In this study, scRNA-seq-based clustering uncovered heterogeneity, including arterial endothelial cell subpopulations enriched for genes involved in quiescence, proliferation and angiogenesis. PAH subpopulations demonstrated a higher contribution of cells with gene enrichment for angiogenesis and proliferation, consistent with known pathological findings in PAH. Whether clusters enriched for angiogenesis corroborate our developmental arterial clusters is unknown. For this investigation, cells were only isolated from the main pulmonary artery and first to fourth branches, whereas our data represent more distal and terminal branches of the developing arterial tree. Nevertheless, comparison of the top genes in the human control and PAH angiogenic clusters with our data do suggest *CXCR4* as a shared gene. *Cxcr4* is enriched in our distal angiogenesis cluster and suggests a common pathway in development and disease.

Our unbiased cell clustering and gene annotation of these clusters identified *Gkn3* and *Alox12* as representative markers of the proximal and distal maECs, respectively. GKN3 was previously identified as an arterial endothelial marker in the brain (Vanlandewijck et al., 2018), indicating its potential as a marker for subpopulations of the arterial endothelium in other vascular beds. ALOX12 has been implicated in the regulation of angiogenesis in other organs and in maladaptive vascular remodeling in pulmonary hypertension (Preston et al., 2006; Zhang et al., 2018; Zheng et al., 2020). This functional prediction is in line with considering ALOX12 as a marker for distal artery endothelium. Similarly, GO analysis of proximal artery endothelium suggested a role in extracellular structure and matrix formation. The importance of our ability to distinguish functional endothelial cellular compartments is twofold. First, we have highlighted genes crucial for angiogenesis that are distinct from those involved in vascular wall assembly. This distinction provides the framework for studying known and novel signaling pathways in a compartmental and functional process to identify potential mechanisms and therapies for vascular regeneration. Second, components of signaling pathways, such as Notch signaling, are broadly expressed in arterial EC populations (Mack and Iruela-Arispe, 2018). Our arterial EC subpopulation gene enrichment analysis now allows us to define differential expression of receptors, ligands and/or transcription factors in these specific arterial subpopulations to improve interrogation of signaling mechanisms. As such, future studies could be designed to examine the interactions of both distal and proximal arterial endothelial cells within their specific niches during vascular growth and assembly.

### CXCL12 signaling defines lung EC compartments and regulates vascular growth

CXCL12 signaling is an evolutionarily conserved angiogenic pathway important for vascular development and regeneration (Red-Horse and Siekmann, 2019). The pathway functions primarily in the recruitment of cells via chemotaxis-directed migration, but it is also implicated in cellular proliferation (Bianchi and Mezzapelle, 2020; Majumdar et al., 2014). Directed cell migration is mediated by a gradient of CXCL12 expression with cellular recruitment to the area of highest expression. One example is the coronary arterial system derived from a vascular plexus that arises from the sinus venosus (Red-Horse et al., 2010). Disruption of this gradient by deletion of CXCL12 results in failure of the coronary artery system to connect to the aorta (Ivins et al., 2015). Whether CXCL12 functions in a similar fashion in the lung is still unclear as we were unable to replicate the full phenotype of the global knockout using EC-specific deletion of CXCL12. Although haptotaxis could still be a mechanism for pulmonary vascular assembly, the source of CXCL12 is likely via ECs and mesenchymal cells for recruitment of ECs to the growing vessel. Studies designed to confirm this hypothesis would have to include a dual Cre recombinase approach using EC-, VSMC- and other mesenchymal-specific Cre recombinases together. Nevertheless, CXCL12 signaling in the endothelium appears to be the primary mechanism behind pulmonary vascular development. Indeed, deletion of CXCR4 using a pan-endothelial Cre recombinase corroborated the branching defects seen with global CXCL12 deletion (Kim et al., 2017). CXCR4 is found on the distal artery and capillary ECs, and ACKR3 is expressed in capillary and venous endothelium. Thus, CXCR4 and ACKR3 expression in their respective compartments could explain the reduction in capillary and venous density observed in *Cxcl12^DsRed/DsRed^* mutants. Future studies deleting these receptors from arterial, capillary or venous endothelium should clarify these mechanisms.

We observed aberrant branching and growth of the pulmonary arterial tree as has been previously described after deletion of CXCL12 (Kim et al., 2017). We have further expanded on this finding and have quantified branching defects seen in *Cxcl12^DsRed/DsRed^* mutants. Furthermore, we discovered a role for CXCL12 in growth of the pulmonary vasculature. Our mutants had significantly reduced arterial and capillary density, suggesting ubiquitous pulmonary vascular hypoplasia. Although branching and the total number of arteries in mutants were increased, the mean diameters were significantly decreased. Smaller vessels will occupy significantly less volume than the larger vessels seen in control embryos and this may explain the reduction in density. Capillary EC deficits have been previously described in an adult pneumonectomy regeneration model in mice. Deletion of CXCL12 in platelets or CXCR4 alone or combined with ACKR3 deletion resulted in a significant reduction in pulmonary capillary EC proliferation, indicating fetal programs as a mechanism for regeneration in the lung (Rafii et al., 2015). Indeed, a CXCL12 therapeutic approach has been used to treat hyperoxia-induced neonatal rodent lung injury (De Paepe et al., 2020; Guerra et al., 2019).

### The CXCL12 endothelial signaling niche affects multiple cell types in the lung

UMAP-based clustering and cell annotation in our scRNA-seq on control versus *Cxcl12^DsRed/DsRed^* mutant embryonic lungs revealed significant cellular population and transcriptomic changes in multiple cell types. Although both ECs and alveolar fibroblasts appeared to be reduced in number, the EC effect was pan-vascular. Differences in the transcriptome occurred in several tissue compartments. In particular, the immune system and epithelium demonstrated significant transcriptomic changes.

For the immune system, CXCL12 and immune cell chemotaxis is well studied. Expression of CXCL12 in the endothelium allows recruitment and transmigration of cells across the vessel wall to areas of infection and inflammation (Cheng et al., 2014). In our data, transcriptomic changes appeared predominantly in alveolar and interstitial macrophages. CXCR4 is broadly expressed by immune cells in our dataset, but ACKR3 is restricted to macrophages. Both receptors are implicated in recruitment of macrophages and differentiation from monocytes (Man et al., 2012; Sánchez-Martín et al., 2011). Despite this evidence, given our global knockout strategy, it is not known whether transcriptional changes in immune cell types are a consequence of early developmental alterations arising in the bone marrow or innate to the lung.

Epithelial-endothelial interactions play an important role in lung development and regeneration. Growth factors such as vascular endothelial growth factor A (VEGFA), Wingless and Int-1 (WNT) ligands and sonic hedgehog (SHH) mediate reciprocal communication to orchestrate both airway and vascular development (Cornett et al., 2013; Del Moral et al., 2006; Peng et al., 2013; Vila Ellis et al., 2020; Yamamoto et al., 2007). Although these interactions are direct in lung development, the mechanism of CXCL12 signaling mediating vascular development is unclear. In our single-cell profiling of lung cells, the epithelium does not express CXCR4, ACKR3 or any additional chemokine receptors in this family. This may be a result of the limitations of scRNA-seq and its sequencing depth as there is other evidence of CXCR4 expression in AT2s in humans (Murdoch et al., 1999). Alternatively, there may be an indirect relationship mediated via an additional niche cell, such as mesenchyme or immune cell populations. Despite these transcriptomic changes, our data revealed no effect on proliferation in the epithelium in null embryonic lungs nor was there a change in the amount of VSM surrounding arteries, indicating that most gross anatomical defects are restricted to the endothelium.

In summary, we have defined a CXCL12 signaling single-cell atlas. In doing so, we uncovered arterial heterogeneity with distinct functional transcriptomes. These findings will improve our understanding of signaling mechanisms along the pulmonary arterial tree. In addition, the CXCL12 signaling axis plays an important role in all developing lung endothelial compartments. As such, CXCL12 signaling may prove to be a therapeutic target for diverse forms of pulmonary vascular hypoplasia and in promoting lung vascular regeneration after injury.

## MATERIALS AND METHODS

### Animals

Development and genotyping information of mouse lines *Cxcl12*^tm2.1Sjm/^J (The Jackson Laboratory, stock #022458, Cxcl12^DsRed^) and *Cxcl12*^tm1.1Sjm/^J (The Jackson Laboratory, stock #022457, Cxcl12^loxp/loxp^) have been previously described (Ding and Morrison, 2013). The endothelial-specific Cre driver *Tek^Cre^* [Tg(*Tek-Cre^1Ywa^*), stock #008863] was used to delete CXCL12 in the endothelium (Kisanuki et al., 2001). Timed matings were performed, counting the day of plug as E0.5. Lungs were harvested from embryos from both heterozygous (*Cxcl12^DsRed/+^*) and homozygous knockout (*Cxcl12^Dsred/DsRed^*) mice, and they were prepared as noted below for whole-mount and tissue section staining. All animal studies were performed in adherence to the guidelines of the Children's Hospital of Philadelphia Institutional Animal Care and Use Committee.

### Whole-mount imaging

Lungs were harvested at E12.5, E13.5, E15.5, E17.5 and E18.5 from both heterozygous (*Cxcl12^DsRed/+^*) and homozygous knockout (*Cxcl12^Dsred/DsRed^*) mice. Based on previously established protocols (Das et al., 2019), lungs were fixed in 2% paraformaldehyde for 4 h and then imaged for DsRed to evaluate the arterial endothelium or prepared for immunofluorescence-based whole-mount imaging. Lung lobes were incubated with primary antibody (rabbit anti-VWF, 1:100, F3520, Sigma-Aldrich; goat anti-EMCN, 1:200, AF4666, R&D Systems), in 5% donkey serum and 0.5% Triton X-100 in PBS (PBST) for 4 h at room temperature (RT) on a shaker, and overnight at 4°C on a shaker. The samples were washed in 0.5% PBST (four 30 min washes at RT), then overnight at 4°C on a shaker. The samples were then incubated in secondary antibody (donkey anti-mouse Alexa Fluor 488, A-21202 and anti-goat Alexa Fluor 647, A21447, Thermo Fisher Scientific, 1:250), in 5% donkey serum and 0.5% PBST for 1 h at RT on a shaker followed by overnight at 4°C. The lungs were washed in 0.5% PBST (four 30 min washes at RT), fixed in 2% paraformaldehyde for 2 h at RT and then washed with PBS (two 30 min washes at RT). Finally, the lungs were cleared using CUBIC (RIA reagent) overnight at 37°C, and then stored at 4°C until imaged using a Leica DMi8 Thunder system and/or Leica DMi8 confocal microscope.

### Immunohistochemistry

Lungs were harvested and fixed as previously described (Frank et al., 2019). They were then washed, dehydrated, embedded in paraffin, and 6-µm sections were obtained. IHC was performed on E15.5, E17.5 and E18.5 sections. In brief, the sections were deparaffinized and rehydrated followed by antigen retrieval (Reveal Decloaker, MSPP-RV1000M, Biocare Medical). The sections were incubated with 3% H_2_O_2_ for 15 min to quench endogenous peroxidases, blocked with 5% donkey serum, and, finally, incubated with primary antibody (rabbit anti-VWF, 1:250, F3520, Sigma-Aldrich; goat anti-EMCN, 1:200, AF4666, R&D Systems; mouse anti-ACTA2, 1:500, A5228, Sigma-Aldrich; rabbit anti-RFP, 1:50, 600-401-379, Rockland Immunochemicals) in 0.1% PBST overnight at 4°C. The presence of relevant proteins was visualized using Alexa Fluor secondary antibodies (donkey anti-mouse Alexa Fluor 488, 1:250, A-21202, Sigma-Aldrich; donkey anti-rabbit Alexa Fluor 555, A-31572, Thermo Fisher Scientific; donkey anti-goat Alexa Fluor 647 1:250, A-21447, Thermo Fisher Scientific).

### RNA FISH

RNA FISH was performed with the RNAscope Multiplex fluorescent V2 Assay [323100, Advanced Cell Diagnostics (ACDBio)] per the manufacture's recommendations. RNAscope 3-plex negative control probe (*DapB*) and mouse specific 3-plex positive control probe (*Polr2a*) were used for control sections. RNAscope target probes included *Cxcl12*, *Cxcr4*, *Ackr3*, *Gkn3*, *Alox12*, *Gja5*, *Nrg1*, *Apln* and *Gpihbp1*, and images were obtained on channels C1, C2, C3 and C4 with Opal fluorophore reagents (Opal 520 FP1487001, Opal 540 FP1494001, Opal 570 FP1488001 or Opal 650 FP1496001, Akoya Biosciences). Finally, slides were incubated in DAPI for 30 s and then mounted using Prolong Gold Antifade Mountant (P36930, Thermo Fisher Scientific).

### Quantification of arterial branching using Imaris Filament

The Filament analysis function of Imaris 9.2 was utilized to trace the arteries using manual tracing with the ‘autopath’ function. The branching angle, number of branches and Sholl analysis was calculated using predetermined algorithms within the function. In brief, the number of branches at the periphery of the lung was calculated after identifying the quaternary branch, which was spatially conserved between *Cxcl12^DsRed/+^* and *Cxcl12^DsRed/DsRed^* mice. To do this, we identified the same region of the lung between various samples and marked the quaternary branch that was conserved between these samples. Sholl analysis involved plotting concentric circles in outward direction at 1 µm distance from the point of origin, and then identifying the number of branches that intersects every concentric circle. Finally, the diameter of all branches was individually measured and were classified as secondary (2°), tertiary (3°), quaternary (4°) and quinary (5°) in the proximal-to-distal direction and based on the hierarchy of arterial branching in the lungs.

### Quantification of vascular density and vein counts

RNA FISH combined with IHC was performed on sections as noted above. Capillary and arterial density were measured on 40× magnification confocal *z*-stacks using Imaris software (version 9.7.2). From whole lung, six identical regions were selected per mice lung. For each image, the whole-tissue region was measured with 3D reconstruction of DAPI-stained tissue using the ‘surface’ function. The total tissue volume was acquired using the ‘volume statistics’ function in Imaris. In order to measure vessel density, a single channel of macrovascular (VWF), capillary (EMCN) or arterial (Cxcl12-DsRed) marker was also reconstructed in 3D using the ‘surface’ function. For capillary density, macrovasculature (VWF) was excluded from the 3D reconstruction to measure only capillaries. The tissue volume was measured using volume statistics. The same threshold parameters were applied when comparing knockout and control groups. Both capillary and arterial density were obtained by dividing the capillary or arterial volume by the total lung volume per image (Chen et al., 2021; Nizari et al., 2019).

Arterial and vein count was measured from 40× magnification confocal images, with six identical regions selected per mouse lung. For each image, vessels stained with an arterial marker (*Gja5* RNA FISH or DsRed) and a macrovascular endothelial marker (VWF) were manually counted. The final vein count was obtained by excluding arteries from the total number of macrovessels stained. The vein count was then divided by the total number of vessels to avoid bias of image selection. Vascular smooth muscle wall thickness was evaluated by IHC for ACTA2, imaged at 40× magnification and measured using Fiji.

### Quantification of proliferation

To quantify proliferative endothelial cells, we first performed IHC for pan-endothelium (rabbit anti-ERG, 1:100, ab214341, Abcam) and EMCN, which stains for capillary and venous endothelium. Following IHC, we performed a click chemistry-based EdU proliferation assay (Click-iT Plus EdU Alexa Fluor 647, C10640, Thermo Fisher Scientific). Pregnant dams were injected intraperitoneally at the desired time point with 50 gm/kg of EdU, and embryos were harvested 4 h following injection. We captured five random areas on each tissue section from each embryo. We counted the percentage of EdU^+^ cells in arteries, veins and capillaries. Briefly, we counted all ERG^+^ cells to determine the total endothelial cells in the field of view. We classified EMCN^−^/ERG^+^ cells as arterial endothelial cells and EMCN^+^/ERG^+^ cells as either capillary or venous endothelial cells. We differentiated venous cells from capillary cells by tracing a subset of cells which formed a lumen. Among these, EdU^+^ cells were counted to determine the percentage of proliferative cells in each subtype.

### Endothelial cell isolation

For the scRNA-seq time course of CXCL12^+^ endothelium, *Cxcl12^DsRed/+^* embryos were harvested at E13.5, E15.5, E18.5 and P8. For control versus knockout lung scRNA-seq comparison, *Cxcl12^DsRed/+^* and *Cxcl12^DsRed/DsRed^* embryos at E18.5 were harvested. Lungs were removed, minced and processed into a single-cell suspension in a Collagenase I (8 mg/5 ml, 17100017, Life Technologies)/Dispase (1:10, 354235, Corning)/RNase-free DNase (1:500, M6101, Promega) solution for 20 min at 37°C (Frank et al., 2016). CD31^+^/TdTomato^+^ cells underwent FACS-based isolation from single-cell suspension using a MoFlo Astrios EQ (Beckman Coulter) flow cytometer with antibody staining for CD31-PECy7 (25-311-82, Thermo Fisher Scientific), CD45-APC (17-0451-82, Thermo Fisher Scientific) and EpCAM- FITC (11-5791-82, Thermo Fisher Scientific). Following negative selection for CD45 and EpCAM, CD31^+^/TdTomato^+^ ECs and CD31^−^/TdTomato^+^ mesenchymal-enriched cells were collected in FACS buffer without EDTA.

### scRNA-seq library preparation and next-generation sequencing

Cells were pelleted and live cell concentration was determined using Trypan Blue staining. The cell pellet was resuspended as per the 10x Genomics recommendation, and 16,500 cells were loaded onto the 10x Chromium Controller (10x Genomics), to target a recovery of 10,000 cells. The libraries were prepared according to the manufacturer's protocol using 10x Single cell 3′ v3 chemistry. These libraries were then sequenced on an Illumina HiSeq 2500 instrument. CellRanger Count v3.1 (10x Genomics) was used to align reads onto the mm10 reference genome.

### Analysis of single-cell sequencing data

For the *Cxcl12* knockout dataset, but not the dataset of *Cxcl12^+^* cells only, ambient background RNA was cleaned from the scRNA-seq data with ‘SoupX’ as described previously (Schuler et al., 2021; Young and Behjati, 2020). The following genes were used to estimate the non-expressing cells, calculate the contamination fraction, and adjust the gene expression counts: *Dcn*, *Bgn*, *Aspn*, *Ecm2*, *Fos*, *Hbb-bs*, *Hbb-bt*, *Hba-a1*, *Hba-a2*, *Lyz1*, *Lyz2*, *Mgp*, *Postn*, *Scgb1a1*. For all datasets, quality filtering was then used to remove cells with >10% or <0.5% mitochondrial mRNA, and to remove cells with <700 detected genes.

Dimensionality reduction, clustering and visualization were performed using Seurat v3.2.2 and sctransform (Hafemeister and Satija, 2019; Stuart et al., 2019), with manual inspection of the expression patterns of the marker genes *Pecam1*, *Ccl21a*, *Vwf*, *Nrg1*, *Plvap*, *Car4* and *Mki67*. sctransform was run with each sequencing reaction as a batch variable with the percentage of mitochondrial RNA as a regression variable. Further data cleaning was performed to remove gene counts for *Gm42418*, which is likely an rRNA (Kimmel et al., 2020). After removal of *Gm42418*, genes with expression in fewer than ten cells across the dataset were removed from further analysis. sctransform (version 0.3.2.9008) was used with glmGamPoi (version 1.2.0) to normalize the data prior to principal component analysis, UMAP embedding, and cell clustering (Ahlmann-Eltze and Huber, 2021). For the *Cxcl12*^+^ sorted dataset, Louvain clustering was performed on the first 25 principal components with a resolution of 0.2. For the dataset investigating the effect of *Cxcl12* knockout, Louvain clustering was performed on the first 20 principal components with a resolution of 0.8. The difference in resolution was due to the number of cell types present in these two different experiments. All charts and heatmaps as part of the scRNA-seq analysis were generated with ggplot2, and all parts of the analysis were run in R 4.0.2. PANTHER GO enrichment analysis (version 10.5281/zenodo.5725227 released 2020-11-01) was performed on the top 40 genes enriched for interrogated clusters and reported by −log10(FDR) (Ashburner et al., 2000; Gene Ontology Consortium, 2021; Mi et al., 2019).

Predictions of RNA velocity were made using a velocyto, scVelo, CellRank pipeline. Spliced and unspliced mRNA counts were determined with velocyto (version 0.17.17) (La Manno et al., 2018) and analyzed using scVelo (version 0.2.3) with the dynamical model (Bergen et al., 2020). CellRank (version 1.5.0) was then used to infer the initial and terminal cell states (Lange et al., 2022). All packages were run in Python 3.7.11. A complete collection of all package versions, and code for all steps of the analysis is available at https://github.com/SucreLab/Cxcl12LungVascularDevelopment.

### Statistical analysis

To avoid assumption of normal distribution of data, nonparametric statistical tests were applied when applicable to test the significance between genotypes. Unpaired *t*-test was applied to compare changes in the number of branches, branching angle and diameter of the filaments. Mann–Whitney *U*-test was applied to detect the significant difference in arterial, venous and capillary density, arterial and venous number, and proliferation changes. Further, for Sholl analysis, Kolmogorov–Smirnov test was performed to detect significance difference. For all the tests, the significance level was set at α=0.05. All statistical tests were performed using GraphPad Prism 9 or MATLAB (2019_b).

## Supplementary Material

10.1242/develop.200909_sup1 Supplementary information
