# Supplementary material for: CXCL12 defines lung endothelial heterogeneity and promotes distal vascular growth
Source: Development. 2022 Oct 31;149(21):dev200909. doi: 10.1242/dev.200909 (PMC9687018; doi:10.1242/dev.200909)
Supplement: Supplementary information [file develop-149-200909-s1.pdf]

Figure S1

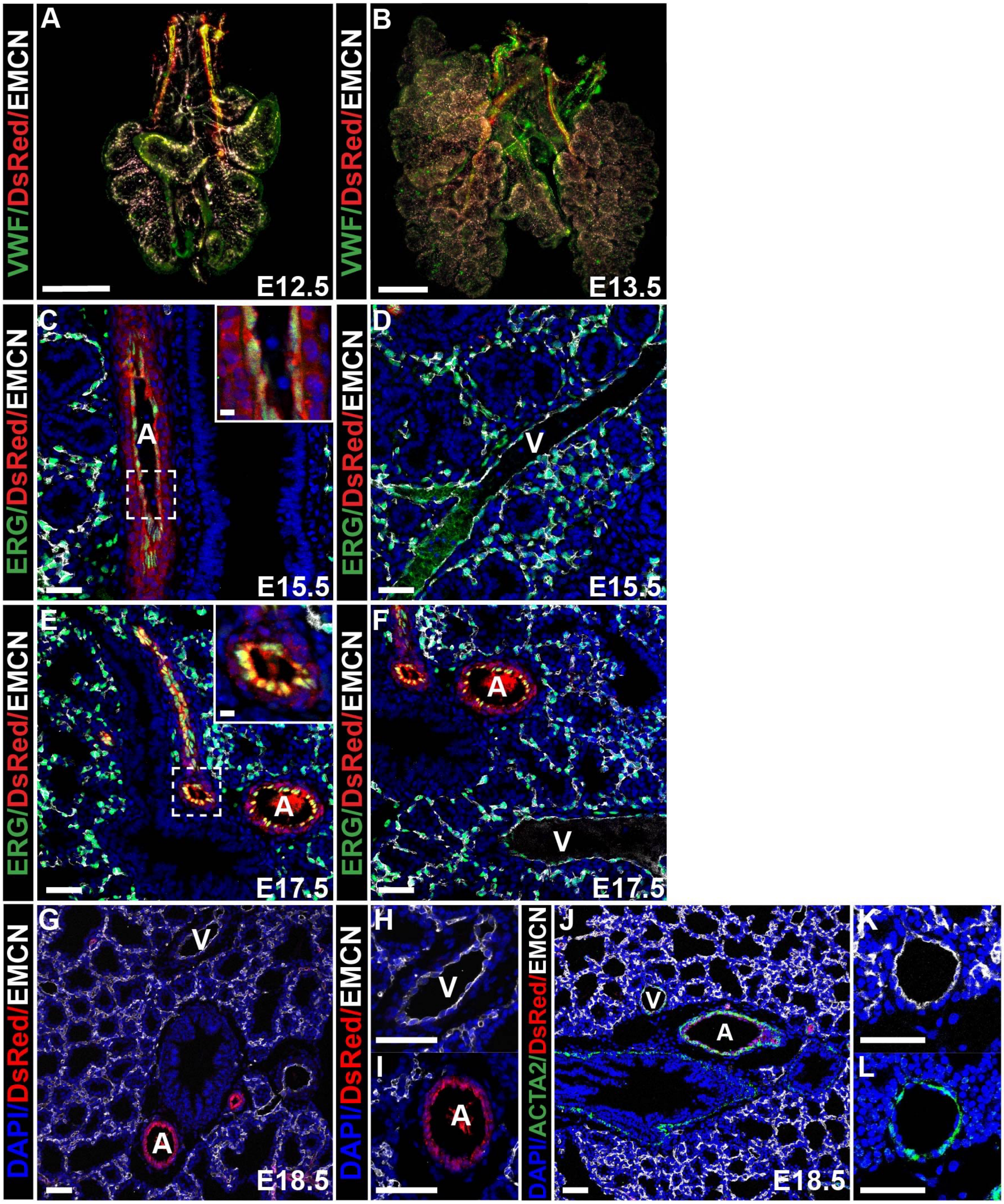
A
VWF/DsRed/EMCN
E12.5
B
VWF/DsRed/EMCN
E13.5
C
ERG/DsRed/EMCN
A
E15.5
D
ERG/DsRed/EMCN
V
E15.5
E
ERG/DsRed/EMCN
A
E17.5
F
ERG/DsRed/EMCN
A
V
E17.5
G
DAPI/DsRed/EMCN
V
A
E18.5
H
DAPI/DsRed/EMCN
V
I
A
J
DAPI/ACTA2/DsRed/EMCN
V
A
E18.5
K
L

Fig. S1. *Cxcl12*-DsRed+ cells in the pulmonary vasculature. (A,B) Whole mount imaging of VWF, DsRed, and EMCN in E12.5 (A) and E13.5 (B) lungs. (C-F) IHC staining for ERG, DsRed, and EMCN at E15.5 and E17.5. (G-I) IHC for DsRed and EMCN to mark arteries (A) and veins (V) at E18.5. (H) Higher magnification of (G) showing EMCN in veins (V). (I) Higher magnification of (G) showing DsRed staining in ECs and VSMCs. (J-L) IHC for DsRed, ACTA2, and EMCN to mark arteries (A) and veins (V) at E18.5. (K,L) Higher magnification of (J) showing EMCN and ACTA2 in veins. Scale bars: A-B = 500 μm, C-L = 50 μm, insets = 10 μm.

Figure S2.

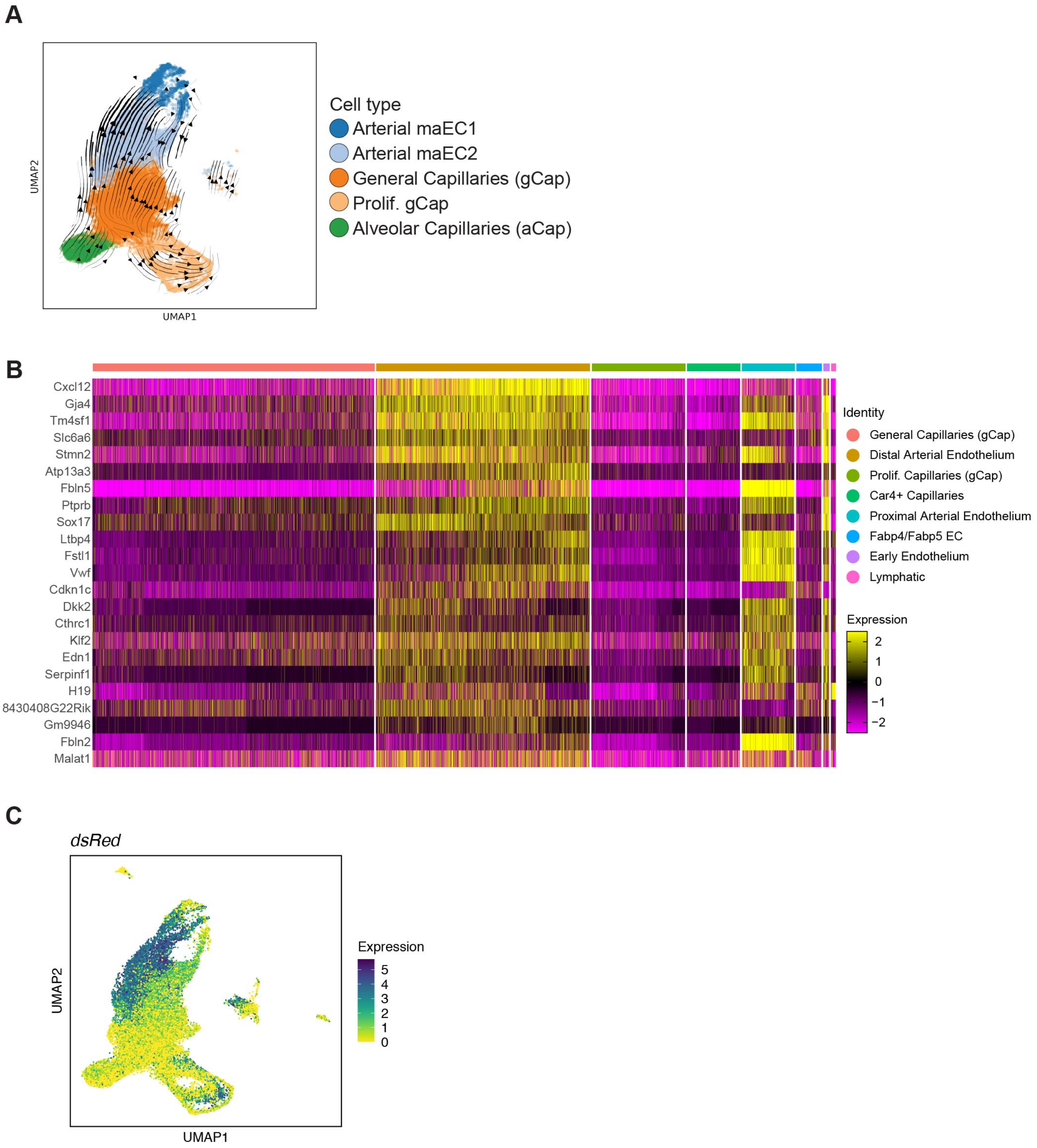

Fig. S2. Predicted RNA velocity, gene expression, and correlation with *Cxcl12*. (A) RNA velocity vectors were calculated with CellRank and overlayed on the UMAP embedding. Thicker lines indicate greater predicted RNA velocity. (B) Heat map of highly correlated genes in all EC populations. (C) UMAP embedding of *Cxcl12*-DsRed+ cells colored by DsRed transcript expression.

Development • Supplementary information

Figure S3

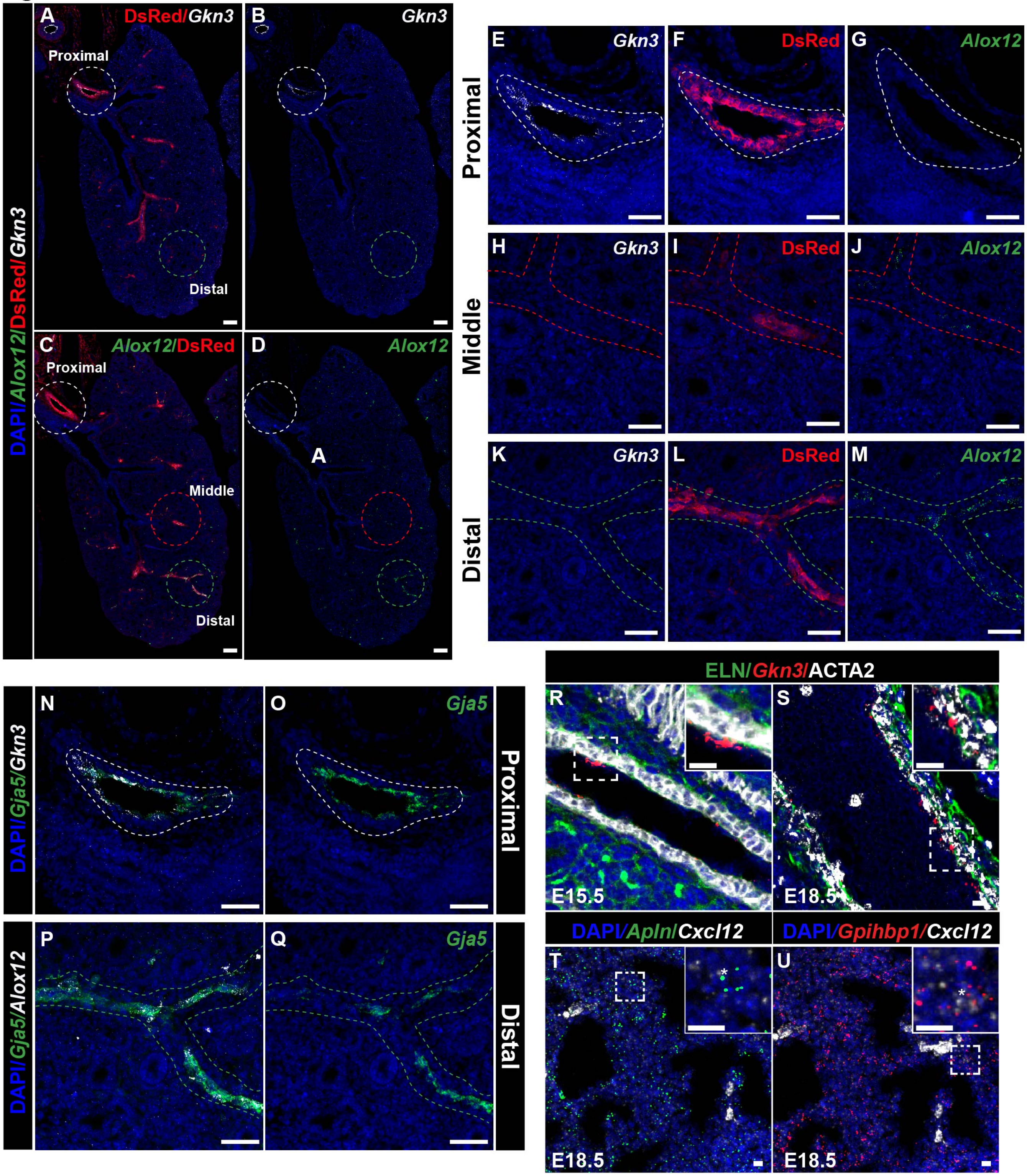

Development • Supplementary information

Fig. S3. Characterization of *Cxcl12*+ ECs in proximal and distal vasculature.
(A-D) RNA FISH for *Gkn3* and *Alox12* and IHC for DsRed protein at E15.5. Dashed circles mark the proximal (white), middle (red), and distal (green) arterial tree. (A) RNA FISH for *Gkn3* and IHC for DsRed protein. (B) Break out channel for *Gkn3* RNA FISH showing expression in the proximal artery. (C) RNA FISH for *Alox12* and IHC for DsRed protein at. (D) *Alox12* channel from (C). (E-M) Higher magnification images from marked regions in (A,B). Break out channels for *Gkn3* (E,H,K) DsRed protein (F,I,L) and *Alox12* (G,J,M) staining. (N,O) RNA FISH for *Gkn3* and *Gja5* at E15.5 in the proximal artery. (P,Q) RNA FISH for *Alox12* and *Gja5* at E15.5 in the distal artery. (R,S) IHC staining for ELN (tropoelastin) and ACTA2 with RNA FISH for *Gkn3* at E15.5 (I) and E18.5 (J). (K,L) RNA FISH for *Cxcl12*, *Apln*, and *Gpihbp1* in the capillaries at E18.5. * marks Cxcl12 expression in capillaries. Scale bars: A-D = 10 µm, insets = 10 µm.

Figure S4

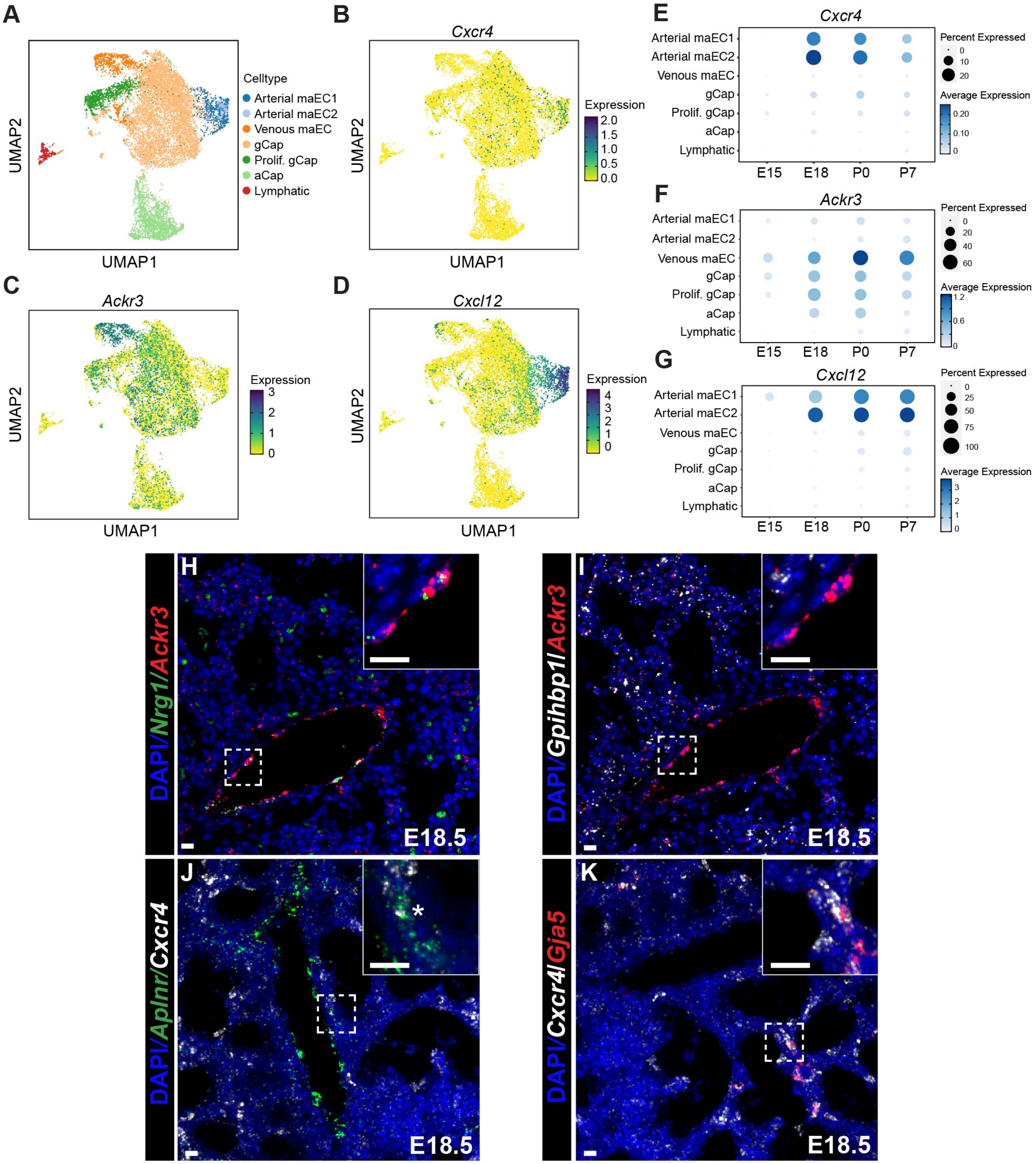

Development • Supplementary information

Fig. S4. Confirmation of *Cxcl12*, *Cxcr4*, and *Ackr3* expression in a whole lung scRNA-seq dataset. (A) UMAP embedding of endothelial cells only, colored by EC populations from publicly available published dataset. (B) UMAP embedding of cells colored by *Cxcr4* expression. (C) UMAP embedding of cells colored by *Ackr3* expression. (D) UMAP embedding of cells colored by *Cxcl12* expression. (E-G) Dot plots for *Cxcr4* (E), *Ackr3* (F), and *Cxcl12* (G) expression in EC populations at various time points. (H-K) RNA FISH for *Cxcr4* and *Ackr3* in capillary and vein ECs. (H,I) RNA FISH for *Ackr3* and *Nrg1* (venous ECs) or Gpihbp1 (capillary ECs) at E18.5. (J,K) RNA FISH for *Cxcr4* and Aplnr (capillary ECs) at E18.5 or *Gja5* (arterial ECs).

* marks capillary expression. Scale bars: H-K = 10 μm, insets = 10 μm.

Development • Supplementary information

Figure S5.

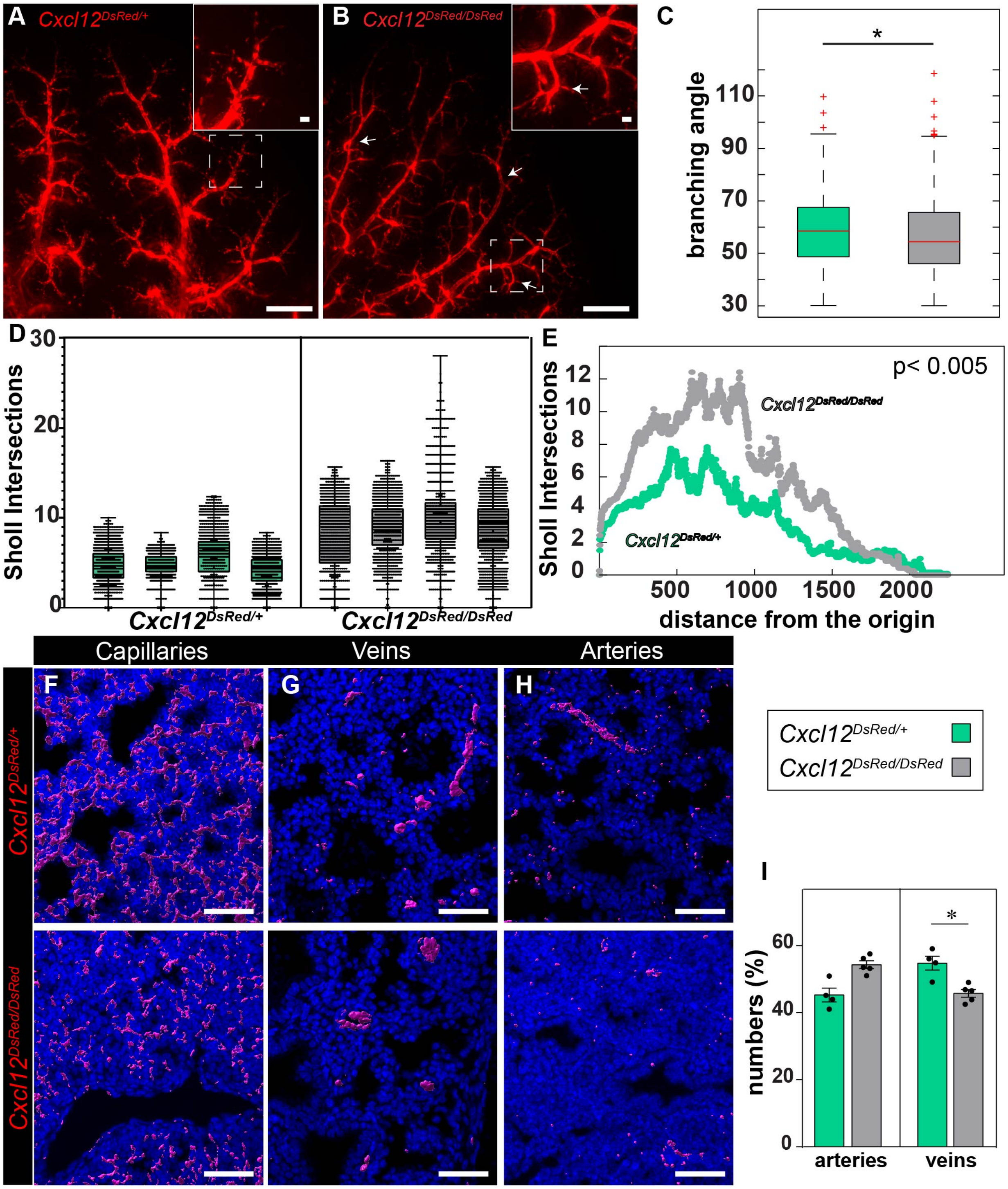

Development • Supplementary information

Fig. S5. Loss of *Cxcl12* leads to aberrant branching and vascular hypoplasia. (A-C) Assessment of peripheral artery branching of *Cxcl12*$^{DsRed/+}$ (A) and *Cxcl12*$^{DsRed/DsRed}$ (B) embryonic lungs. Insets: Higher magnification of distal arteries marked by dashed white boxes. (C) Comparative branching angle measurements of *Cxcl12*$^{DsRed/+}$ versus *Cxcl12*$^{DsRed/DsRed}$ arteries (n=4, Statistical significance: *p<0.05). (D) Sholl analysis showing distribution of each sample. (E) Scatter plot of the average Sholl intersections at all distances from the origin of artery in *Cxcl12*$^{DsRed/+}$ versus *Cxcl12*$^{DsRed/DsRed}$ embryonic lungs (n=4, Statistical significance: *p<0.05). (F-H) Representative volumetric analysis of capillaries (F), veins (G), and arteries (H) using Imaris 3D surface rendering (red represents rendered vessels) in *Cxcl12*$^{DsRed/+}$ (upper panels) versus *Cxcl12*$^{DsRed/DsRed}$ (bottom panels) embryonic lungs. (I) Arterial and Venous percentage in *Cxcl12*$^{DsRed/+}$ versus *Cxcl12*$^{DsRed/DsRed}$ embryonic lungs (n≥4, Statistical significance: *p<0.05). Scale bars: A-B = 500 μm, insets = 50 μm F-H = 50 μm.

Development • Supplementary information

Figure S6.

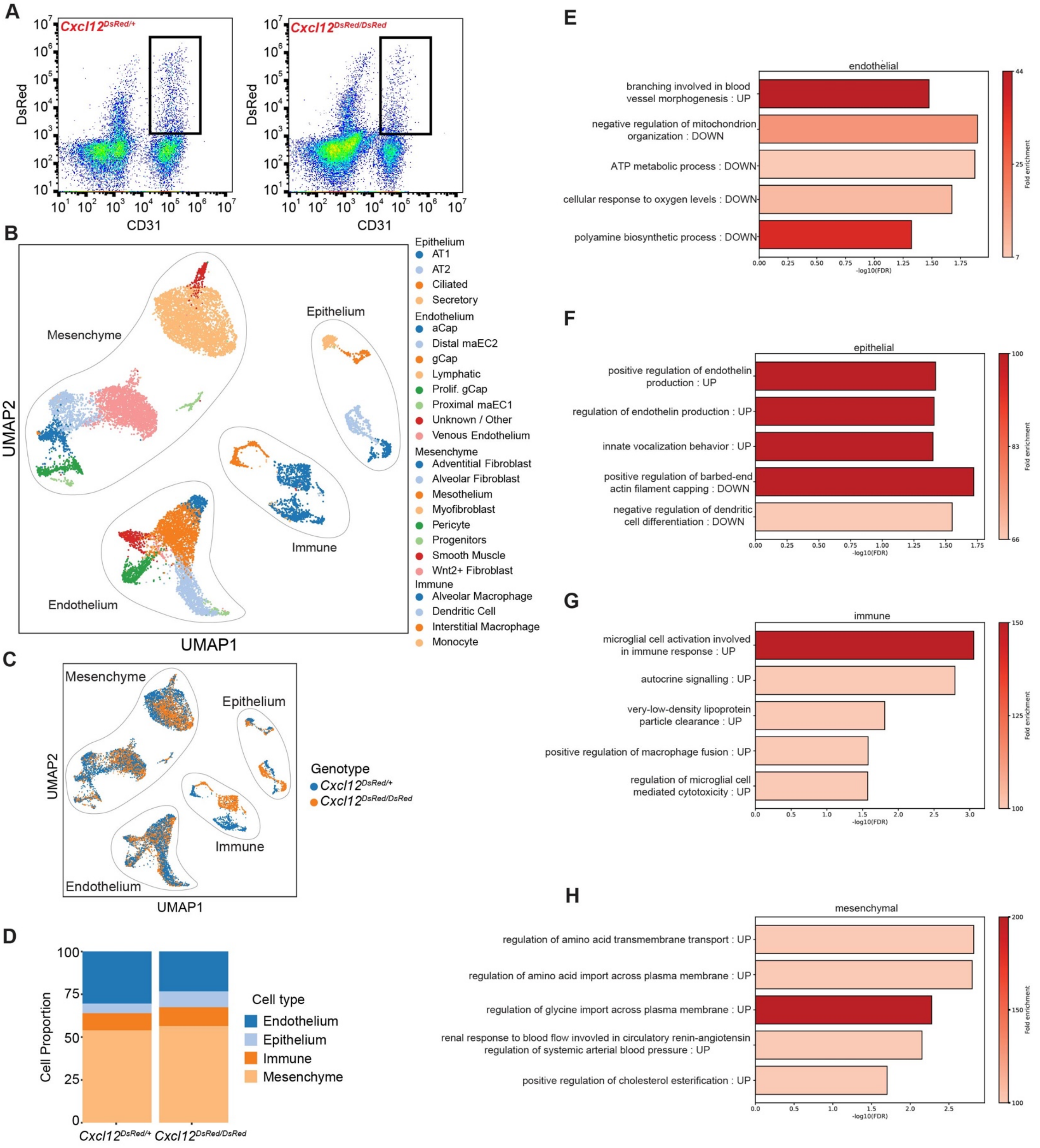

Development • Supplementary information

Fig. S6. scRNA-seq analysis reveals global transcriptome changes in *Cxcl12*$^{DsRed/DsRed}$ whole lungs. (A) FACS plots showing fluorescent intensity of DsRed+ ECs in *Cxcl12*$^{DsRed/+}$ and *Cxcl12*$^{DsRed/DsRed}$ embryonic lungs at E18.5. (B) Integrated *Cxcl12*$^{DsRed/+}$ and *Cxcl12*$^{DsRed/DsRed}$ UMAP embedding of cells colored by cell type to compare *Cxcl12*$^{DsRed/+}$ versus *Cxcl12*$^{DsRed/DsRed}$ embryos at E18.5. (C) UMAP embedding of whole lung cells colored by genotype to compare *Cxcl12*$^{DsRed/+}$ versus *Cxcl12*$^{DsRed/DsRed}$ embryos at E18.5. (D) Changes in cell type proportion between *Cxcl12*$^{DsRed/+}$ and *Cxcl12*$^{DsRed/DsRed}$ embryonic lungs. (E-H) PANTHER GO analysis for top 5 ranked categories using either upregulated (UP) or downregulated gene enrichment sets in cell populations. (E-H) GO analysis for endothelial (E), epithelial (F), immune (G) cells, and mesenchymal (H) cells.

Development • Supplementary information

Figure S7

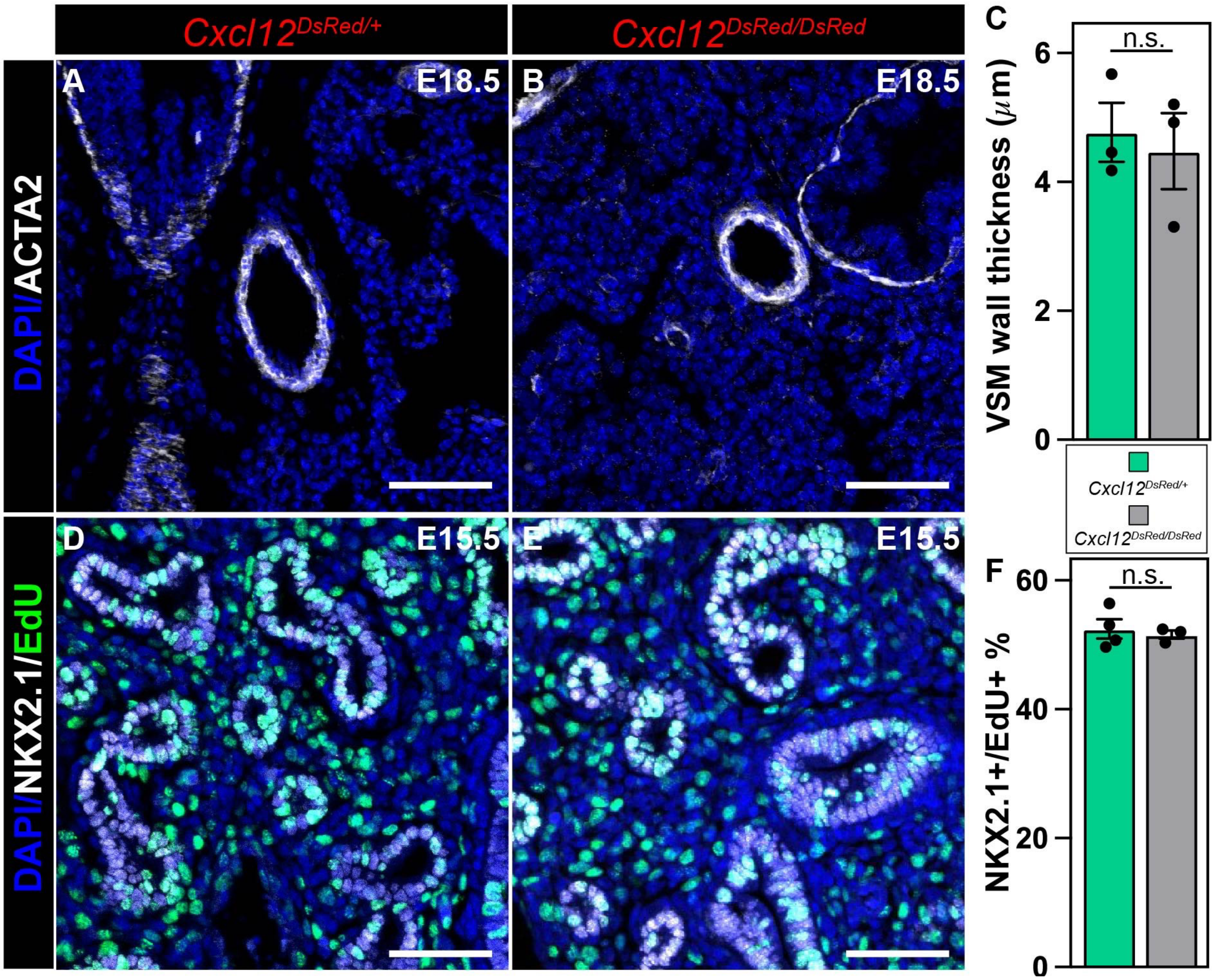

Fig. S7. Effects of global knockout of CXCL12 on the epithelium and VSM. (A,B) IHC for ACTA2 to mark smooth muscle in *Cxcl12*$^{DsRed/+}$ (A) versus *Cxcl12*$^{DsRed/DsRed}$ (B) in E18.5 lungs. (C) Quantification of arterial VSM thickness in *Cxcl12*$^{DsRed/+}$ and *Cxcl12*$^{DsRed/DsRed}$ ($n \geq 3$, $p > 0.99$) lungs at E18.5. (D,E) IHC for NKX2.1 and EdU staining in *Cxcl12*$^{DsRed/+}$ and *Cxcl12*$^{DsRed/DsRed}$ lungs at E15.5. (F) Quantification of epithelial proliferation in *Cxcl12*$^{DsRed/+}$ (D) versus *Cxcl12*$^{DsRed/DsRed}$ (E) lungs at E15.5 measured by EdU incorporation ($n \geq 3$, $p = 0.86$). Scale bars: A, B, D, E = 50 μm.

Development • Supplementary information

Table S1. Enriched genes for cell annotated clusters.

Click here to download Table S1

Table S2. Total GO terms for gene enrichment sets for maEC1s and maEC2s.

Click here to download Table S2

Table S3. Enriched genes for Cxcl12 KO vs WT cell annotated clusters.

Click here to download Table S3

Table S4. Enriched genes used for GO term analysis in Cxcl12 KO vs WT lungs.

Click here to download Table S4
